# Visceral Artery Revascularization With the "Roof-Top" Approach: An Alternative Technique With Great Exposure of the Suprarenal Aorta

**DOI:** 10.7759/cureus.53782

**Published:** 2024-02-07

**Authors:** Konstantinos G Seretis, Theofanis Papas, Afroditi Antoniou, Sofia Tzamtzidou, Andreas M Lazaris

**Affiliations:** 1 Department of Vascular Surgery, Korgialenio-Benakio Hellenic Red Cross Hospital, Athens, GRC; 2 Department of Vascular Surgery, Attikon University Hospital, Athens, GRC

**Keywords:** suprarenal aorta, chronic mesenteric ischemia, roof-top approach, aorto-coeliac bypass, bilateral subcostal incision

## Abstract

Due to the extensive collateral arterial network, symptomatic chronic mesenteric ischemia is a relatively uncommon condition and is associated with severe atherosclerotic disease of all major visceral arteries. Open surgical repair has been commonly used to restore blood supply to the visceral arteries, and the "roof-top" approach has been advocated as an alternative technique to traditional midline incision, mainly because of the great exposure of the suprarenal aorta that it offers. Roof-top approach, in other words, bilateral subcostal incision, is a totally abdominal approach to the suprarenal aorta, and as the title says, it is like a roof-top on the abdominal wall. We present a case of a female patient with intestinal angina that was deemed unsuitable for endovascular repair (ER) and was treated with open surgical repair utilizing the "roof-top" approach.

## Introduction

Chronic mesenteric ischemia (CMI) is caused by occlusive disease of the visceral arteries that are responsible for the blood supply of the gastrointestinal tract. Although occlusive disease of the visceral arteries is a quite common finding in the elderly population, CMI is less common because of the extensive collateral network in the mesenteric circulation [1]. Symptoms generally appear when at least two of the visceral arteries are involved, and surgical treatment is required to reverse this condition and improve quality of life [2]. Open repair (OR) has been advocated as the golden standard in the treatment of CMI and provides immediate relief of symptoms in most patients [3]. Endovascular treatment offers an alternative modality for the elderly and higher-risk patients and recently has surpassed open surgery in the treatment of patients presenting with symptoms of CMI [4]. In the modern era, OP is reserved for patients who have lesions unsuitable for stenting, and evidence from the literature suggests that symptom relief is similar for open and endovascular approaches [5]. However, the choice of therapy is individualized and is based mainly on perceived clinical risk and surgeon preference. 

We present a case of a female patient with symptomatic CMI, and her anatomy was unsuitable for endovascular stenting; therefore, she was referred to us for surgical bypass. The presented case focuses mainly on the technical notes of the "roof-top" approach, an alternative technique that should be included in every vascular surgeon’s quiver dealing with pathologies of the suprarenal aorta [6].

## Case presentation

**Figure 1:**
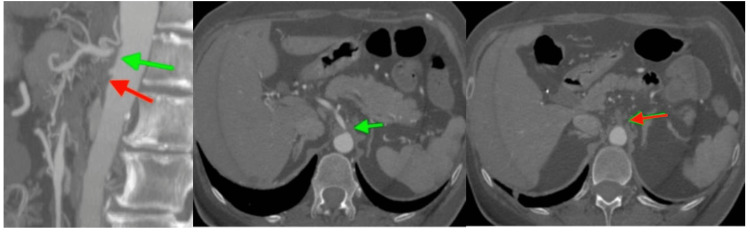
CTA of the abdominal aorta, showing ostial occlusion of the coeliac artery (green arrow) and occlusion of the SMA at its origin (red arrow). CTA, computed tomography angiography; SMA, superior mesenteric artery

Our patient presented with postprandial intestinal angina and reported a 20 kg weight loss in a period of two months. Computed tomography angiography (CTA) of the abdominal aorta revealed total occlusion of all visceral arteries of the patient. The coeliac trunk was occluded at its origin, and superior and inferior mesenteric arteries presented with long thromboembolic lesions that were unsuitable for endovascular treatment (Figure 1).

The original plan was to conduct an antegrade bypass from the aorta to the superior mesenteric artery (SMA), but intraoperatively we decided to perform an endarterectomy to the SMA and an antegrade bypass from the aorta to the coeliac trunk. We preferred to utilize the "roof-top" approach for the wide access to the suprarenal aorta that would grant access to the targeted vessels easier.

**Figure 2:**
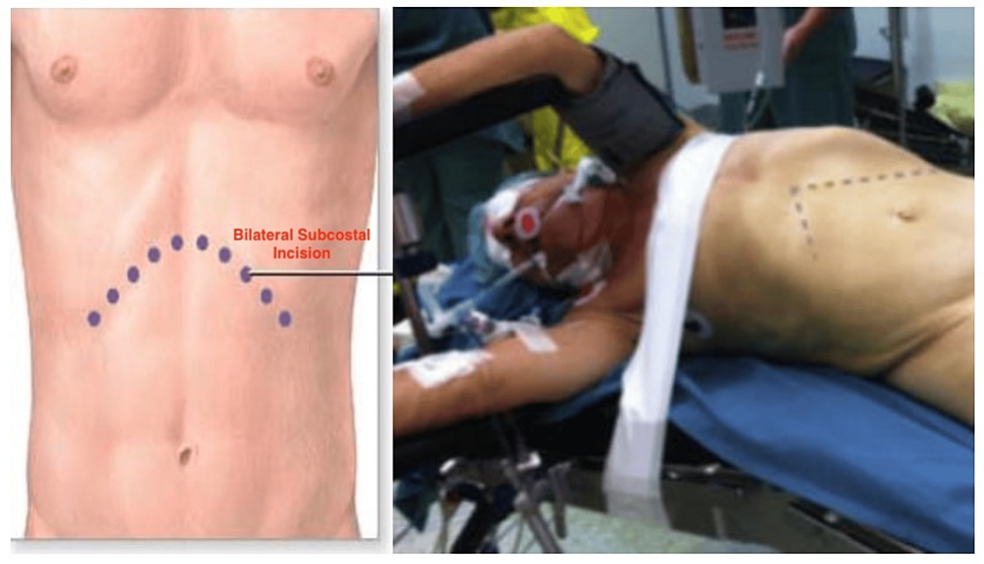
Bilateral subcostal incision and patient positioning in "roof-top" approach (photograph of another patient, used for illustrational reasons to depict positioning).

Our patient was placed in a modified right lateral decubitus position with her shoulders rotated at 60-80^o^ from horizontal and the hips almost horizontal to the operating table. Her left arm was secured on a standing stool across her body, and the operating table was set at a reverse jackknife position. An abdominal bilateral subcostal incision was carried out, starting from the right side just above the right rectus abdominis muscle and extending to the left side down to the left anterior axillary line (Figure 2).

In the midline, the round ligament was ligated and then divided. After the abdominal incision, the lower flap of the incision was everted and tacked down on the right lower quadrant with a heavy suture (i.e., Vicryl No 1), running from the fascia of the flap to the skin of the right lower abdominal wall. 

After entrance to the peritoneal cavity, the sigmoid colon was pulled gently toward the patient’s midline, and the left parabolic groove of Toldt was recognized. Using a pair of Metzenbaum scissors, the peritoneum was sharply divided just at the border of the sigmoid colon and the parietal peritoneum until the sigmoid colon was totally mobilized. A left-side retroperitoneal dissection was then performed parallel to the descending colon, keeping the dissection plane at the posterior of the gonadal vessels, left ureter, kidney, and Gerota’s fascia. This dissection plane was carried upward by blunt mobilization up to the diaphragm. This was followed by a left median visceral rotation: spleen, stomach, pancreas, and left kidney were rolled en bloc to the patient’s right side. The splenophrenic ligament had to be sharply divided prior to rotation in order to avoid capsular tear of the spleen and make everything roll easily to the patient’s right. The left crus of the diaphragm that encompassed the superior part of the abdominal aorta had to be cut in order to allow the supraceliac aorta to be dissected free. The descending lumbar vein, a branch of the left renal vein that is always there, was securely ligated and divided. At this stage, the abdominal aorta had been exposed at its full extent from the diaphragm to the aortic bifurcation, and systemic heparinization followed. The aorta was then clamped in an anteroposterior fashion at a level over the coeliac artery, and an antegrade bypass from the aorta to the coeliac trunk and a thromboendarterectomy to the SMA in standard fashion followed. 

**Figure 3:**
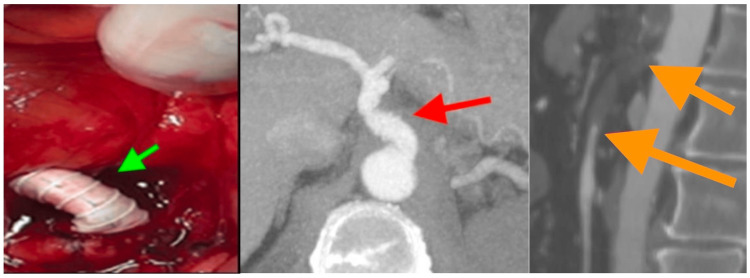
Intraoperative picture showing antegrade bypass from the aorta to the coeliac trunk (green arrow), and CTA of abdominal aorta one month postoperatively, showing patency of the aorta-coeliac bypass graft (red arrow), and recurrent thrombosis of the SMA (orange arrows).

At follow-up, one month after the initial operation, the patient reported complete resolution of symptoms and a 5 kg weight gain. CTA at follow-up showed patency of the aorto-coeliac bypass graft, but SMA was thrombosed, probably because our patient had stopped taking her anticoagulant therapy (Figure 3). 

## Discussion

CMI is a rare disease, caused by atherosclerotic disease of the visceral arteries that leads to stenosis or occlusion of the mesenteric arteries. OR has been considered the gold standard in the treatment of CMI since its first described successful use in 1958, and provides immediate relief of symptoms in the majority of patients [3]. Patients suffering from CMI, because of the atherosclerotic nature of the disease, are often old with concomitant coronal artery disease and atherosclerotic lesions in other vascular beds. Recent advancements in endovascular repair (ER) of CMI since its first described successful application in the form of percutaneous angioplasty of the mesenteric arteries in 1980 have shifted the course of treatment to more minimally invasive procedures [7]. Mesenteric artery stenting is indicated for short lesions without severe calcification, and longer lesions with extensive calcification are not indicated for ER [8]. However, there are many published reviews that reported that ER has lower primary patency when compared to OR, and higher rates of restenosis, symptom recurrence, and secondary interventions are attributed to ER [5,9]. A systemic review published recently comparing ER and OR reported improved primary patency and more durable control of symptoms in patients treated with OR but similar secondary patency for both groups of patients [2]. It has also been suggested that OR has improved long-term outcomes when a repair to both coeliac artery (CA) and SMA is performed [10,11]. Therefore, OR continues to have an important role in the modern era and is reserved mainly for good-risk patients with long lesions and severe calcification. However, there is a tendency to avoid antegrade aorto-coeliac or aorto-superior mesenteric bypasses in high-risk patients, and iliac-based repair with single-SMA bypass is preferred for high-risk patients that are not suitable for ER.

When we need to perform antegrade bypass from the suprarenal aorta to the visceral arteries, the traditional midline transperitoneal approach is challenging, since the exposure is limited at this level [12]. The thoracoabdominal incision could serve as an alternative but is mainly required for thoracoabdominal aneurysms, which extend to the descending thoracic aorta, and is accompanied by greater morbidity (especially pulmonary) when compared with transabdominal approaches. The retroperitoneal approach is considered a more feasible alternative, but when more extended exposure to the level of the supraceliac aorta is needed, entrance into the left thoracic cavity cannot be avoided [13]. The "Roof-top" approach serves as an alternative technique for the treatment of pathologies of the visceral arteries at a suprarenal level, offering great exposure without the need to extend the incision into the left hemothorax [14]. When compared with the retroperitoneal approach, the roof-top approach avoids the thoracic incision while providing essentially the same extent of aortic mobilization. Roof-top approach also offers the advantages of medial visceral rotation and provides adequate exposure to manage complex arterial lesions of the upper abdominal aorta and its branches, but caution must be made in order not to create low perfusion in the viscera by compressing or occluding the SMA and to a lesser extent the coeliac trunk. On the other side, medial visceral rotation allows only restricted exposure of the right side of the aorta and the right iliac artery is totally inaccessible and carries the risk of injury to the pancreas, the spleen, and the visceral vessels. 

In summary, OR continues to have an important role and remains a durable option when performed with low mortality in experienced centers. "Roof-top" is an approach that every vascular surgeon treating pathologies of the visceral arteries should be familiar with, especially when there is a need to perform antegrade bypasses from the aorta to the coeliac and mesenteric arteries. However, the choice of treatment should always be individualized and should be based on patient comorbidities, physician preference, and experience, in order to achieve the best result for the benefit of the patient. 

## Conclusions

CMI is a rare syndrome of atherosclerotic nature that affects mainly elderly patients with multiple comorbidities. OP has its designated place in the modern era and is indicated for selected cases. Antegrade bypasses from the aorta to the visceral arteries are indicated for lower-risk patients, while iliac-based bypasses are offered to higher-risk patients. When an antegrade bypass is indicated, the "roof-top" approach offers great exposure to the suprarenal aorta. This technique is of extreme value and should be included in every vascular surgeon’s armamentarium that is treating various aortic pathologies above the level of the renal arteries. Future research to monitor morbidity and mortality rates of patients undergoing arterial reconstructions with the traditional transabdominal or retroperitoneal approach compared with the roof-top approach is needed to establish treatment guidelines for specific pathologies of the suprarenal aorta.
